# Shear Damage Simulations of Rock Masses Containing Fissure-Holes Using an Improved SPH Method

**DOI:** 10.3390/ma16072640

**Published:** 2023-03-27

**Authors:** Shuyang Yu, Xuekai Yang, Xuhua Ren, Jixun Zhang, Yuan Gao, Tao Zhang

**Affiliations:** 1 School of Transportation and Civil Engineering, Nantong University, Nantong 226019, China; 2 College of Water Conservancy and Hydropower Engineering, Hohai University, Nanjing 210098, China

**Keywords:** SPH, fissures and holes, crack propagation, fracture mechanics, numerical simulation

## Abstract

Fissures and holes widely exist in rock mechanics engineering, and, at present, their failure mechanisms under complex compress and shear stress states have not been well recognized. In our work, a fracture mark, *ξ*, is introduced, and the kernel function of the smoothed-particle hydrodynamics (SPH) is then re-written, thus realizing the fracture modelling of the rock media. Then, the numerical models containing the fissures and holes are established, and their progressive failure processes under the compress and shear stress states are simulated, with the results showing that: (1) the improved SPH method can reflect the dynamic crack propagation processes of the rock masses, and the numerical results are in good agreement with the previous experimental results. Meanwhile, the improved SPH method can get rid of the traditional mesh re-division problems, which can be well-applied to rock failure modeling; (2) the hole shapes, fissure angles, fissure lengths, fissure numbers, and confining pressure all have great impacts on the final failure modes and peak strengths of the model; and (3) in practical engineering, the rock masses are in the 3D stress state, therefore, developing a high performance 3D SPH program and applying it to engineering in practice will be of great significance.

## 1. Introduction

**Figure 1:**
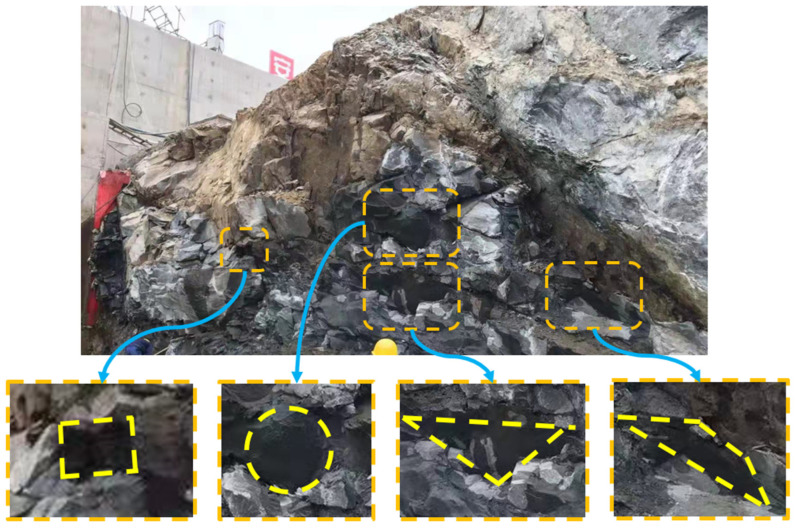
Different types of defects in a typical rock slope.

Rock mass is a typical heterogeneous, anisotropic material that contains large amounts of joints, fissures, holes, and other various forms of defects [1,2]. Figure 1 shows a typical rock slope containing various defects, including complex joints, square holes, circular holes, triangular holes, and trapezoidal holes. The existence of these defects greatly reduces the strength of the rock masses, and the instability of the rock mass engineering will occur under these complex boundary conditions or disturbances, which poses a serious threat to the safety of people’s lives and properties nearby [3]. Therefore, an understanding of the failure mechanisms under this combination of fissures and holes will undoubtedly have important practical significances for preventing and controlling rock engineering disasters.

Present studies on the crack propagation of rock masses mainly concentrate on three aspects: (1) theoretical studies; (2) experimental studies; and (3) numerical simulation. Theoretical studies can quantitatively express the formula of the damage evolution. For example, Baud et al. [4] proposed an improved calculation model for the prediction of the crack propagation directions of rock masses under the compressive stress state; Eftekhari et al. [5] improved the maximum shear stress criterion and quantitatively analyzed the influences of the size and shape of the rock specimens on the crack propagation directions; and Kawamoto et al. [6] established a damage evaluation model for fractured rock masses, according to the damage mechanics. However, theoretical studies can only derive exact solutions for the conditions of the relatively simple boundaries and defect shapes, while complex geometries, as well as boundary conditions, will lead to extremely complex mathematical expressions which cannot be solved. Experimental studies can directly reflect the macroscopic laws of the crack propagation of rock masses. For example, Sagong et al. [7] carried out a uniaxial compression test on rock specimens with prefabricated fissures, and these crack propagation processes were recorded; Yang et al. [8] carried out a uniaxial compression test on the failure processes of sandstone specimens with different fissure lengths and inclination angles, and discussed the effects of these fissure properties on the specimen strength and elasticity modulus; and Lajtai et al. [9] prefabricated a single crack in gypsum specimens and carried out uniaxial compression tests, with their progressive failure processes being obtained. However, experimental studies cannot reveal the internal mechanisms of rock damage processes.

Numerical simulation is a new technique that has been developed in recent years, which can directly reflect the internal mechanisms of rock fracture, which is regarded as the “third method” of scientific studies. The finite element method (FEM) [10] is one of the earliest methods that was used in rock damage simulations; however, it relies heavily on mesh grids. Mesh refinements should be carried out for the discontinuous properties such as cracks or holes. During the crack propagation, the connections and crosses of the cracks need the mesh redivisions, requiring large amounts of calculation resources and potentially even leading to a calculation failure [11,12]. The discrete element method (DEM) can solve the mesh problems that exist in the traditional FEM method, and its computational domain is discretized by various particles, which can easily simulate rock fracture processes [13,14,15]. However, DEM has many mesoscopic parameters. Recently, many new numerical methods have been developed to simulate these rock failure progresses, for example: The PeriDynamics (PD) method [16,17], the Numerical Manifold Method (NMM) method [18,19], and the element-free Galerkin method [20,21], which all have unique advantages, but also have limitations. SPH is a pure Lagrange method that gets rid of the mesh problems in FEM. Meanwhile, SPH does not have many meso-parameters like DEM. The General Particle Dynamics (GPD) method, proposed by Zhou’s groups, has been well-applied to rock fracture mechanics [22,23,24,25,26,27,28,29,30]; however, no SPH works have been focused on hole–fissure interactions.

In this work, a fracture mark, *ξ,* is introduced, and the kernel function of SPH is then re-written, thus realizing the fracture modelling of the rock media. The SPH numerical models under the compress and shear stress states are established and the interactions between the fissures and holes are simulated. These research results can provide some references for the understanding of the fracture mechanisms of the interactions between the rock fissures and holes.

## 2. Basic Principles of SPH Method

### 2.1. SPH Discrete Strategy

If the field function *f*(*x*) inside the integral domain *Ω* is defined and continuous, then it can be exactly expressed in integral form [31]:

(1) $$f(x)=\int_{\Omega }f(x^{\prime })\delta (x-x^{\prime })dx^{\prime }$$

where ***x*** is the coordinate vector; ***x***’ is the coordinate vector of a particular point; *f*(***x***) is the field function, which stands for particle mass, density, energy, and velocity, etc.; *Ω* is the integral domain; and *δ*(***x*** − ***x***’) is the Dirac functions.

Then, in the SPH method, the Dirac delta distribution is replaced by another kernel function, which can be re-written as:

(2) $$f(x)\approx \int_{\Omega }f(x^{\prime })W(x-x^{\prime },\;h)dx^{\prime }$$

### 2.2. Particle Approximation

In the SPH method, the calculation system is composed of a finite number of particles with independent masses, which occupy an independent space. The field function, or its derivative that is approximated by the kernel function, can be converted into a discrete form of a superposition and the summation of all the particles in its support domain, which can be expressed as follows [32]:

(3) $$f(x_{i})=\sum_{j=1}^{N}\frac{m_{j}}{\rho_{j}}f(x_{j})W_{ij}\;$$

where *i* and *j* are the SPH particle sequence numbers. Equally, we can obtain the particle approximation formula for the derivative of the field function:

(4) $$\nabla f(x_{i})=\sum_{j=1}^{N}\frac{m_{j}}{\rho_{j}}f(x_{j})\nabla W_{ij}\;$$

### 2.3. Governing Equations

Here, in SPH, four governing equations must be obeyed, the first of which is the density equation, which describes the updating of the particle density; the second is the momentum equation, which describes the updating of the particle velocity; the third is the energy equation, which describes the updating of the particle energy; and the last is the motion equation, which expresses the updating of the particle position. The four governing equations can be expressed as [31]:

(5) $$\left\{\begin{array}{c} \frac{d\rho_{i}}{dt}=\sum_{j=1}^{N}m_{j}v_{ij}^{\beta }\frac{\partial W_{ij,\beta }}{\partial x_{i}^{\beta }}\\ \frac{dv_{i}^{\alpha }}{dt}=\sum_{j=1}^{N}m_{j}(\frac{\sigma_{i}^{\alpha \beta }}{\rho_{i}^{2}}+\frac{\sigma_{j}^{\alpha \beta }}{\rho_{j}^{2}}+T_{ij})\frac{\partial W_{ij,\beta }}{\partial x_{i}^{\beta }}\\ \frac{de_{i}}{dt}=\frac{1}{2}\sum_{j=1}^{N}m_{j}(\frac{\sigma_{i}^{\alpha \beta }}{\rho_{i}^{2}}+\frac{\sigma_{j}^{\alpha \beta }}{\rho_{j}^{2}}+T_{ij})v_{ij}^{\beta }\frac{\partial W_{ij,\beta }}{\partial x_{i}^{\beta }}\\ \frac{dx_{i}^{\alpha }}{dt}=v_{i}^{\alpha } \end{array}\right.$$

where *ρ* represents the particle density; *t* stands for the time step; *m* represents the particle mass; *v* stands for the particle velocity, *v_ij_* means *v_i_*–*v_j_*; *x* represents the particle position; *σ_αβ_* stands for the stress components; *W* is the so-called smoothing kernel function; and *T* represents the artificial viscosity.

## 3. Damage Model in SPH Method

### 3.1. Damage Criterion

The SPH particle failure criterion adopts the Mohr–Coulomb criterion with a tension cut off, which is expressed in the following form [33]:

(6) $$\sigma_{n}=\sigma_{t}$$

(7) $$\tau_{f}=c+\sigma_{f}\tan\varphi$$

In Equations (7) and (8), *σ_n_* and *σ_t_* represent the normal stress and tensile strength, respectively; *τ_f_* stands for the shear stress on the failure surface; *c* represents the particle cohesion, while *φ* stands for the internal friction angle.

### 3.2. Damage Treatments in SPH Method

As can be derived from Equation (6), the derivative of *W* determines the updates of the key parameters in SPH. Then, we can find that the progressive deterioration of the rock masses can be realized by adding a fracture state mark to *W*. Here, a fracture mark, *ξ*, is introduced, and the derivative of the kernel function can then be improved as follows [25]:

(8) $$\frac{\partial D_{ij,\beta }}{\partial x_{i}^{\beta }}=\xi_{i}\frac{\partial W_{ij,\beta }}{\partial x_{i}^{\beta }}$$

**Figure 2:**
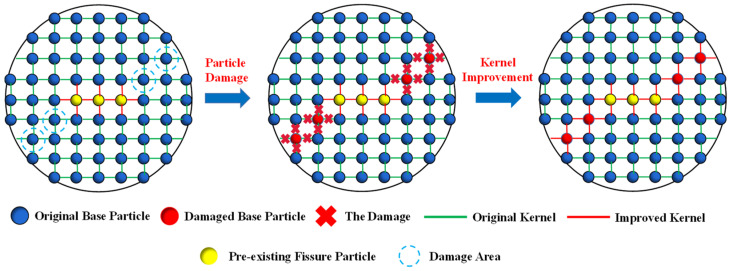
Particle damage treatments.

In Equation (8), *D* stands for the improved form of *W*. The value of the fracture mark, *ξ,* can be illustrated as below: when the stress on a particular particle reaches the damage criterion, then the *ξ* is set to be 0; otherwise, *ξ* is equal to 1. The surrounding kernels have been changed, which then leads to a change of the target particles. Therefore, the particle damage treatments can be clearly exhibited in Figure 2. The numerical implementations of the improved SPH method are based on the Fortran language and the Visual Studio platform, which are revised from Liu’s open-source program.

## 4. SPH Model and Calculation Conditions

### 4.1. Parameter Calibrations

As discussed earlier, rock is a typical heterogeneous material, and, in order to characterize the heterogeneity of rock masses, the two-parameter Weibull function [34] is introduced in this section, which has been widely accepted in the previous literature to characterize the spatial variability of the compressive strength of rock masses. The expressions of the two-parameter Weibull distribution can be listed below:

(9) $$f(x)=\frac{m}{x_{0}}{\left(\frac{x}{x_{0}}\right)}^{m-1}\exp\left[-{\left(\frac{x}{x_{0}}\right)}^{m}\right]$$

In Equation (9), *x* stands for the mechanical properties of a particular particle, including the elasticity modulus and compressive strength, etc.; *m* is the heterogeneity coefficient, representing the heterogeneity of the rock masses; and *x*_0_ stands for the mean value of the basic particle parameters.

**Figure 3:**
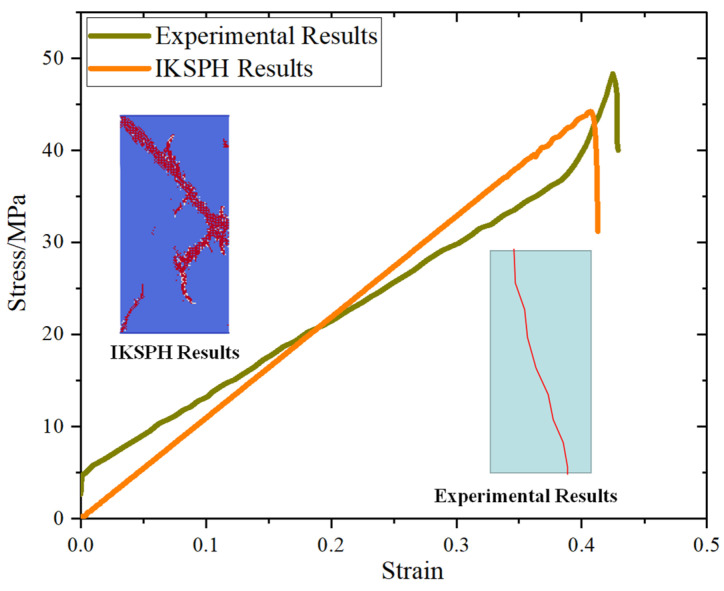
Comparisons between numerical results and experimental results [35].

Figure 3 shows the stress–strain curves that were obtained with the SPH method and their comparisons with previous experimental results [35]. The failure mode of the SPH results is the shear failure, whose failure surfaces are from the top to the bottom. The stress–strain curves are similar, and the bedding error at the beginning may be the testing errors of the experiments. We can find that these numerical results are consistent with the previous experimental results, which means that the calibrated parameters can then be used for simulation.

### 4.2. SPH Model of Rock Specimen Containing Fissure-Holes

**Figure 4:**
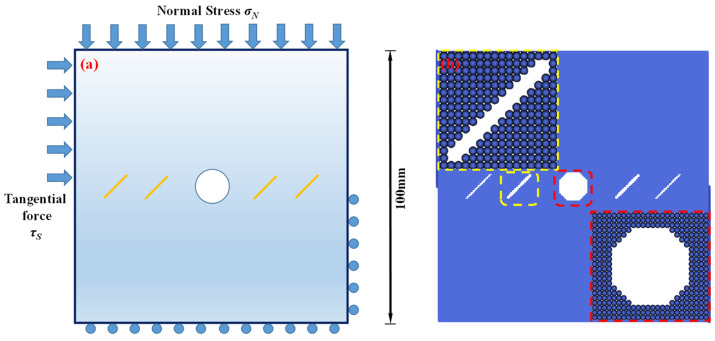
Calculation model and particle divisions. (**a**) Calculation model and boundaries; and (**b**) particle divisions.

In order to investigate the interactions between the holes and fissures under the compress and shear stress states, the fissure–hole SPH numerical model is established in this section, which is shown in Figure 4. The model size is set as 100 mm × 100 mm, and the diameter of the hole is 12 mm. In this model, one hole is set in the center of the model, and the fissures are prefabricated at the hole sides. The vertical pressure *σ_N_* is applied at the top of the model, and the tangential force *τ_S_* is applied at the upper left side of the model. The lower right side and the bottom side are the fix boundaries, as shown in Figure 4a. The whole model is divided into 200 × 200 = particles. The model mechanical parameters are listed below: elastic modulus *E* = 17 GPa, Poisson’s ratio *μ* = 0.2, *m* = 5. What should be stressed is that all the simulations in the current study use 2D plane stress conditions.

### 4.3. SPH Calculation Conditions

In actual rock mechanics engineering, there are often combinations of different fissure forms and different hole shapes. In this section, the following calculation conditions are set to reflect these various conditions: A: different hole shapes, such as rectangle holes, circular holes, triangle holes, and trapezoid holes; B: different fissure angles, such as *θ* = 15°, 45°, 60°, and 75°; C: different fissure numbers, such as *N* = 2, 4, 6, and 8; and D: different fissure lengths, such as *l* = 6 mm, 12 mm, 18 mm, and 24 mm. The details are shown in Table 1.

## 5. SPH Simulation Results

### 5.1. Failure Mode Analysis of Fissure-Hole Interactions

**Figure 5:**
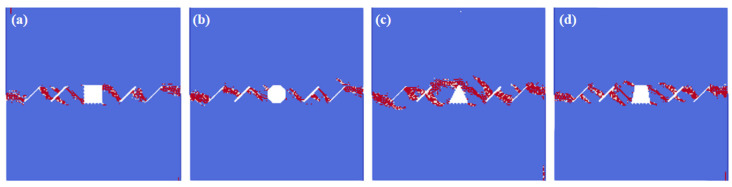
The interactions of holes and fissures under different hole shapes. (**a**) Rectangle hole; (**b**) circular hole; (**c**) triangle hole; and (**d**) trapezoid hole.

Figure 5 shows the crack interaction laws under the different hole shape conditions. In this model, the white color represents the tensile failure, and the red color represents the shear failure. We can see from the numerical results that: under the actions of compress and shear stress, the failure mode is mostly the shear failure. The fissures connect to each other, and the crack also propagates from the hole to the fissures, leading to the failure of the whole model. The different hole shapes also have impacts on the interactions between the hole and the fissures. The interaction locations of the rectangle hole are in the directions of 2 o’clock and 8 o’clock, which fall into the corner of the square. The interaction locations of the circular hole are in the directions of 3 o’clock and 9 o’clock. All three corners of the triangle holes connect with the pre-existing fissures. The interaction locations of the trapezoid hole are in the directions of 2 o’clock and 6 o’clock, and the cracks of the other two corners of the trapezoid hole initiate.

**Figure 6:**
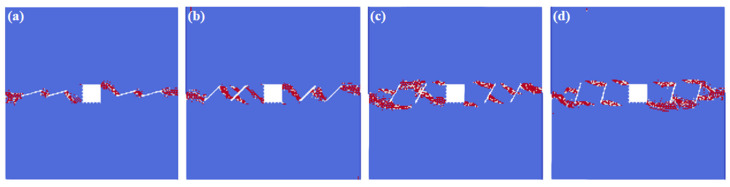
The interactions of holes and fissures under different fissure angles. (**a**) *θ* = 15°; (**b**) *θ* = 45°; (**c**) *θ* = 60°; and (**d**) *θ* = 75°.

Figure 6 shows the crack interaction laws under the different fissure angle conditions. We can infer from the numerical results that: the fissure angles have great influences on the interactions between the hole and the fissures. When the fissure angle is relatively small (*θ* = 15°), the fissures connect to each other, but the interaction locations change from the original square corners to the square sides. When the fissure angle is *θ* = 45°, cracks initiate not only from the fissure tips, but also from the fissure middle. However, the cracks initiating from the fissure middle stop the propagation if they extend to a certain extent, and the final failure mode is the cracks which initiate from the fissure tips that propagate through the model. When the fissure angle is *θ* = 60°, most of the cracks initiate from the middle of the fissures, rather than from the fissure tips, and the final failure mode is the cracks which initiate from the fissure middle that propagate through the model. What should be noticed is that the interaction locations in this condition are different from the previous conditions, which mainly concentrate in the directions of 5 o’clock, 7 o’clock, and 11 o’clock. When the fissure angle is *θ* = 75°, the initiations of the fissures that are closer to the rectangle hole mainly occur on the fissure tips; however, the initiations of the fissures further away from the rectangle hole happen both at the fissure tips and in the middle. The interaction locations of the rectangle hole are in the 5 o’clock and 11 o’clock directions in this condition.

**Figure 7:**
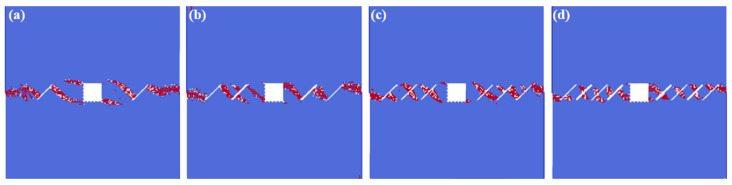
The interactions of holes and fissures under different fissure numbers. (**a**) *N* = 2; (**b**) *N* = 4; (**c**) *N* = 6; and (**d**) *N* = 8.

Figure 7 exhibits the rock’s crack interaction laws under the different fissure numbers. As can be inferred from the simulation results: the fissure numbers have impacts not only on the fissure interaction laws, but also on the fissure–hole interaction modes. For the condition where the fissure number is relatively small (*N* = 2), the cracks initiate from the fissure tips, and the interaction locations fall into the directions of 2 o’clock and 7 o’clock. Meanwhile, the cracks of the rectangle hole itself also initiate, which fall in the directions of 5 o’clock and 11 o’clock. For the condition where the fissure number is *N* = 4, the fissures that are closer to the rectangle hole initiate at the fissure middle. For the conditions of *N* = 6 and *N* = 8, the fissures are more likely to initiate from the fissure middle.

**Figure 8:**
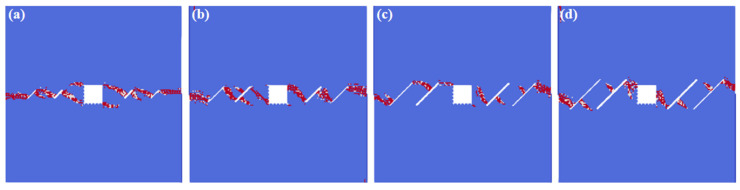
The interactions of holes and fissures under different fissure lengths. (**a**) *l* = 6 mm; (**b**) *l* = 12 mm; (**c**) *l* = 18 mm; and (**d**) *l* = 24 mm.

Figure 8 shows the interaction laws under the different fissure lengths. We can infer that, when the fissure length is relatively small (*l* = 6 mm), the cracks initiate from the fissure tips. However, with the increase in the fissure lengths, the cracks gradually initiate from the middle of the fissures. What should be pointed out is that, when the fissure length is relatively small, the interaction locations of the rectangle hole are in the square corner. However, with the increase in the fissure length, the interaction locations gradually move to the square sides.

**Figure 9:**
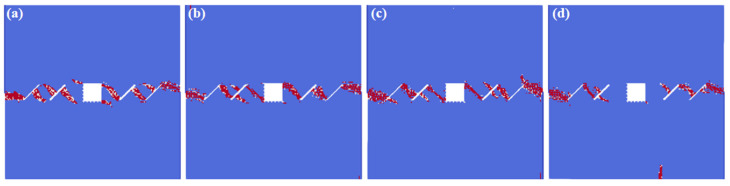
The interactions of holes and fissures under different vertical stress. (**a**) *σ_N_* = 0.5 MPa; (**b**) *σ_N_* = 1 MPa; (**c**) *σ_N_* = 1.5 MPa; and (**d**) *σ_N_* = 2 MPa.

Figure 9 shows the rock’s crack interaction laws under the different confining pressures. We can see the increase in the vertical stress typical “inhibition” effect on the density of the crack generations, which means that the cracks are more difficult to initiate and propagate. What is worth noticing is that, when the vertical stress reaches 2 MPa, under the same calculation steps, the cracks do not run through the model.

### 5.2. Analysis of Initiation and Failure Pressures

**Figure 10:**
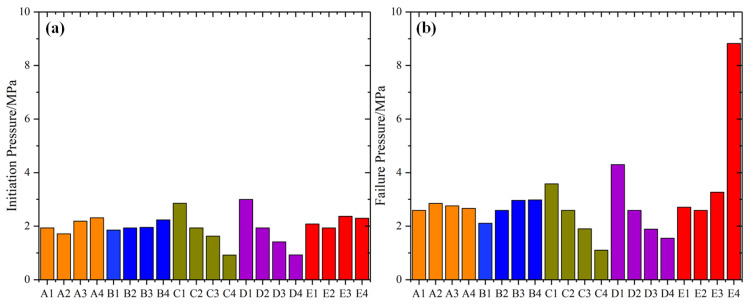
The initiation and failure pressure. (**a**) Initiation pressure; and (**b**) failure pressure.

Figure 10 exhibits the initiation and failure pressures under the different conditions. We can see that: for the different hole shapes, the initiation pressure reaches its lowest in condition A2 (circular hole), and reaches its highest in condition A4 (trapezoid hole), while the failure pressure is just the opposite, which indicates that the circular holes lead to the ductility failure, while the trapezoidal holes lead to the brittle failure. With the increase in the fissure angle, the initiation and failure pressure increase accordingly, but the differences between the different fissure angles are relatively small. The increase in the fissure numbers and lengths has a strong reduction effect on the initiation and failure pressure of the model, which indicates that the fissure numbers and lengths are the important factors that affect the rock strength. In general, the increase in the confining pressure increases the model’s shear strength, and what should be noticed is that the failure pressure under the condition of σN = 2 MPa increases sharply compared with the other conditions, which means that the confining pressure of 2 MPa may be a threshold value.

## 6. Discussion

### 6.1. Rock Crack Propagation Morphology

**Figure 11:**
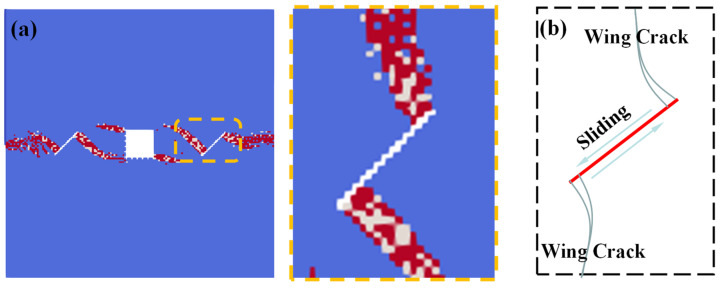
“Wing crack” morphology. (**a**) “Wing crack” propagation calculated by SPH method; and (**b**) previous experimental results [35].

We can see from Figure 11 that: for single fissure under the compress and shear stress states, the tensile stress concentrates at the fissure tips due to the relative dislocations of the fissure surfaces, thus leading to the formations of the so-called “wing cracks”. Figure 11 also shows the comparisons between the numerical results of condition C1 and the previous experimental results [35]. The “wing crack” initiates from the fissure tips and propagates along the loading direction, which is consistent with the previous experimental results.

**Figure 12:**
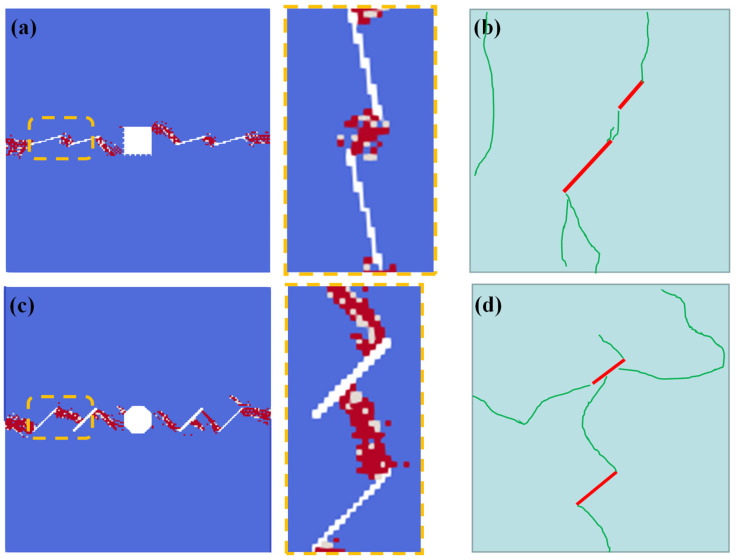
Comparisons of multiple crack interaction laws between SPH results and previous experimental results. (**a**) Numerical results of condition B1; (**b**) previous experimental results 1 [36]; (**c**) numerical results of condition A2; and (**d**) previous experimental results 2 [37].

For multiple fissures, the crack morphology can be divided into two categories, according to the relative locations of the pre-existing fissures: one condition is that the fissure tips are close to each other, and the “wing crack” directly connects with them, which forms a big crack. The numerical results of condition B1 are shown in Figure 12a, which are consistent with the previous experimental results [36], as shown in Figure 12b. Another condition is when the fissure tips are far away. In this condition, the “wing crack” initiates from the fissure tips, propagates, and connects to another fissure middle. The numerical results of condition A2 are shown in Figure 12c, which are consistent with the previous experimental results, as shown in Figure 12d.

### 6.2. Initiation Laws of Different Hole Shapes

**Figure 13:**
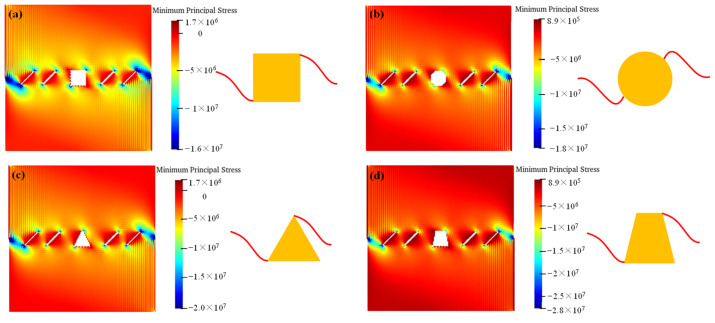
Distributions of maximum principal stress under different hole shapes. (**a**) Maximum principal stress of condition A1; (**b**) maximum principal stress of condition A2; (**c**) maximum principal stress of condition A3; and (**d**) maximum principal stress of condition A4.

We can infer from Section 5 that the hole shapes also have impacts on the interactions between the holes and the fissures. In order to quantitatively characterize the mechanical properties around the hole, the distributions of the maximum principal stress under the different hole shapes are shown in Figure 13. As can be seen, the shear stress concentration leads to the crack initiation and propagation. For the rectangle hole, the shear stress concentrates in the directions of 2 o’clock and 7 o’clock; for the circular hole, the shear stress concentrates in the directions of 2 o’clock and 7 o’clock; for the triangle hole, the shear stress concentrates in the directions of 7 o’clock and 12 o’clock; and for the trapezoid hole, the shear stress concentrates in the directions of 1 o’clock and 7 o’clock. The interaction locations in Section 4 all agree well with the shear stress concentration areas. Therefore, the internal mechanisms of the complex hole–fissure interactions can be quantitatively reflected by the stress distributions in SPH. 

However, 2D SPH simulations are carried out in this work, while 3D simulations would be closer to the real situations. Therefore, future research should focus on the 3D high-performance SPH parallel programs.

## 7. Conclusions

(1) The rock fracture properties can be realized with the SPH method by adding a fracture mark, *ξ*, to multiply it with the traditional kernel function.
(2) Different hole–fissure numerical models have been established and simulated. “Wing cracks” initiate from the fissure tips, and the interaction locations between the holes and the fissures are at the hole corners. The numerical results are verified by comparisons with the previous experimental results.
(3) The circular hole has the most reduction on the specimen strength, while the trapezoid hole has the least. The failure strength increases with an increase in the fissure angle.
(4) The fissure lengths and numbers are the two key factors that influence the peak strength of rock masses. Meanwhile, an increase in the confining pressure also increases the shear strength of the specimen.

## Figures and Tables

**Table 1:** Calculation conditions of the rock model with different fissures and holes.

Model	Condition	Details	Model	Condition	Details
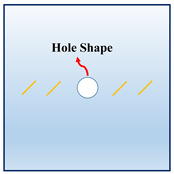	A1	Rectangle Hole	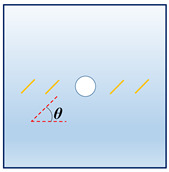	B1	*θ* = 15°
	A2	Circular Hole		B2	*θ* = 45°
	A3	Triangle Hole		B3	*θ* = 60°
	A4	Trapezoid Hole		B4	*θ* = 75°
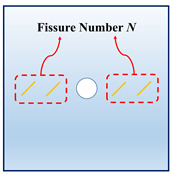	C1	*N* = 2	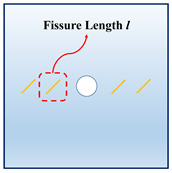	D1	*l* = 6 mm
	C2	*N* = 4		D2	*l* = 12 mm
	C3	*N* = 6		D3	*l* = 18 mm
	C4	*N* = 8		D4	*l* = 24 mm
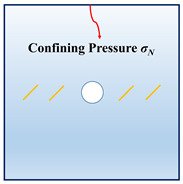			E1	*σ_N_* = 0.5 MPa	
			E2	*σ_N_* = 1 MPa	
			E3	*σ_N_* = 1.5 MPa	
			E4	*σ_N_* = 2 MPa	

## Data Availability

Data will be available upon reasonable request.

## References

[1] Zhang W.B., Shi D.D., Shen Z.Z., Wang X.H., Gan L., Shao W., Tang P., Zhang H.W., Yu S.Y. (2023). Effect of calcium leaching on the fracture properties of concrete. Constr. Build. Mater..

[2] Zhang W.B., Shi D.D., Shen Z.Z., Shao W., Gan L., Yuan Y., Tang P., Zhao S., Chen Y.S. (2023). Reduction of the calcium leaching effect on the physical and mechanical properties of concrete by adding chopped basalt fibers. Constr. Build. Mater..

[3] Deng P., Liu Q., Huang X. (2021). Acquisition of normal contact stiffness and its influence on rock crack propagation for the combined finite-discrete element method (FDEM). Eng. Fract. Mech..

[4] Baud P., Reuchlé T., Charlez P. (1996). An improved wing crack model for the deformation and failure of rock in compression. Int. J. Rock Mech. Min. Sci. Geomech. Abstr..

[5] Eftekhari M., Baghbanan A., Hashemolhosseini H. (2016). Crack propagation in rock specimen under compressive loading using extended finite element method. Arab. J. Geosci..

[6] Kawamoto T., Ichikawa Y., Kyoya T. (1988). Deformation and fracture behaviour of discontinuous rock mass and damage mechanics theory. Int. J. Numer. Anal. Methods Geomech..

[7] Sagong M., Bobet A. (2002). Coalescence of multiple flaws in a rock-model material in uniaxial compression. Int. J. Rock Mech. Min. Sci..

[8] Yang S., Jing H. (2011). Strength failure and crack coalescence behavior of brittle sandstone samples containing a single fissure under uniaxial compression. Int. J. Fract..

[9] Lajtai E. (1974). Brittle fracture in compression. Int. J. Fract..

[10] Yang S., Cao M., Ren X. (2018). 3D crack propagation by the numerical manifold method. Comput. Struct..

[11] Zhao Q., Du J., Huang Z. (2020). Study on Crack Propagation Characteristics of Pitch Bearing Based on Sub-model. J. Phys. Conf. Ser..

[12] Branco R., Antunes F., Costa J. (2015). A review on 3D-FE adaptive remeshing techniques for crack growth modelling. Eng. Fract. Mech..

[13] Hadjigeorgiou J., Esmaieli K., Grenon M. (2009). Stability analysis of vertical excavations in hard rock by integrating a fracture system into a PFC model. Tunn. Undergr. Space Technol..

[14] Ding X., Zhang L. (2014). A new contact model to improve the simulated ratio of unconfined compressive strength to tensile strength in bonded particle models. Int. J. Rock Mech. Min. Sci..

[15] Amin M., Mohammad F. (2012). Numerical analysis of confinement effect on crack propagation mechanism from a flaw in a pre-cracked rock under compression. Acta Mech. Sin..

[16] Shou Y., Zhou X., Qian Q. (2016). Dynamic Model of the Zonal Disintegration of Rock Surrounding a Deep Spherical Cavity. Int. J. Geomech..

[17] Zhou X., Shou Y. (2016). Numerical Simulation of Failure of Rock-Like Material Subjected to Compressive Loads Using Improved Peridynamic Method. Int. J. Geomech..

[18] Ohnishi Y., Sasaki T., Koyama T. (2014). Recent insights into analytical precision and modelling of DDA and NMM for practical problems. Geomech. Geoengin..

[19] Miki S., Sasaki T., Koyama T. (2010). Development of coupled discontinuous deformation analysis and numerical manifold method (NMM–DDA). Int. J. Comput. Methods.

[20] Tunsakul J., Jongpradist P., Soparat P., Kongkitkul W., Nanakorn P. (2014). Analysis of fracture propagation in a rock mass surrounding a tunnel under high internal pressure by the element-free Galerkin method. Comput. Geotech..

[21] Tunsakul J., Jongpradist P., Kim H., Nanakorn P. (2018). Evaluation of rock fracture patterns based on the element-free Galerkin method for stability assessment of a highly pressurized gas storage cavern. Acta Geotech..

[22] Zhou X., Zhao Y., Qian Q. (2015). Smooth particle hydrodynamic numerical simulation of rock failure under uniaxial compression. Chin. J. Rock Mech. Eng..

[23] Zhao Y., Zhou X., Qian Q. (2015). Progressive failure processes of reinforced slopes based on general particle dynamic method. J. Cent. South Univ..

[24] Zhou X., Zhao Y., Qian Q. (2015). A novel meshless numerical method for modeling progressive failure processes of slopes. Eng. Geol..

[25] Bi J. (2016). The Fracture Mechanisms of Rock Mass Under Stress, Seepage, Temperature and Damage Coupling Condition and Numerical Simulations by Using the General Particle Dynamics (GPD) Algorithm.

[26] Bi J., Zhou X. (2017). A Novel Numerical Algorithm for Simulation of Initiation, Propagation and Coalescence of Flaws Subject to Internal Fluid Pressure and Vertical Stress in the Framework of General Particle Dynamics. Rock Mech. Rock Eng..

[27] Bi J., Zhou X., Qian Q. (2016). The 3D Numerical Simulation for the Propagation Process of Multiple Pre-existing Flaws in Rock-Like Materials Subjected to Biaxial Compressive Loads. Rock Mech. Rock Eng..

[28] Bi J., Zhou X. (2015). Numerical Simulation of Zonal Disintegration of the Surrounding Rock Masses Around a Deep Circular Tunnel Under Dynamic Unloading. Int. J. Comput. Methods.

[29] Zhou X., Bi J., Qian Q. (2015). Numerical Simulation of Crack Growth and Coalescence in Rock-Like Materials Containing Multiple Pre-existing Flaws. Rock Mech. Rock Eng..

[30] Zhou X., Bi J. (2016). 3D Numerical Study on the Growth and Coalescence of Pre-existing Flaws in Rocklike Materials Subjected to Uniaxial Compression. Int. J. Geomech..

[31] Wang T., Wang J., Zhang P. (2020). An Improved Support Domain Model of Smoothed Particle Hydrodynamics Method to Simulate Crack Propagation in Materials. Int. J. Comput. Methods.

[32] Libersky L.D., Petschek A.G., Carney T.C., Allahdadi F.A. (1993). High strain Lagrangian hydrodynamics a three-dimensional SPH code for dynamic material response. J. Comput. Phys..

[33] Yang S., Chen R. (2023). A new strategy for 3D non-persistent crack propagation by the numerical manifold method with tetrahedral meshes. Eng. Anal. Bound. Elem..

[34] Weibull W. (1951). A statistical distribution function of wide applicability. ASME J. Appl. Mech..

[35] Liu L., Li H., Li X. (2020). Full-field strain evolution and characteristic stress levels of rocks containing a single pre-existing flaw under uniaxial compression. Bull. Eng. Geol. Environ..

[36] Zhu D., Chen Z., Xi J. (2017). Interaction between offset parallel cracks in rock. Chin. J. Geotech. Eng..

[37] Cheng J. (2017). Experimental Study on Crack Propagation Characteristics of Prefabricated Double-Fissure Rock-like Materials under Biaxial Compression.

